# A Comparison of Intrathecal Dexmedetomidine and Fentanyl as Adjuvants to 0.5% Hyperbaric Levobupivacaine for Lower Abdominal Surgeries: A Prospective, Double-Blinded, Randomized Controlled Trial

**DOI:** 10.7759/cureus.76292

**Published:** 2024-12-23

**Authors:** Pranjal Gupta, Ravindra S Chouhan, Krishan G Jangir, Vikram S Rathore, Prakash C Audichya, Sameer Goyal

**Affiliations:** 1 Department of Anaesthesiology and Critical Care, Pacific Medical College and Hospital, Udaipur, IND; 2 Department of Anaesthesiology and Critical Care, Uttar Pradesh University of Medical Sciences, Etawah, IND

**Keywords:** analgesia, dexmedetomidine, fentanyl, hemodynamic, levobupivacaine

## Abstract

Background

Due to its affordability and ease of application, the subarachnoid block is the most frequently used method for lower abdominal procedures. Levobupivacaine has an onset of sensory and motor blockade comparable to that of bupivacaine and prolongs the duration of analgesia while facilitating quick recovery from motor block. Fentanyl and dexmedetomidine, when used as additives to intrathecal local anesthetic, can extend the duration of sensory and motor blockade and enhance postoperative analgesia.

Aims and objectives

The purpose of this study is to examine and compare the effectiveness of fentanyl and dexmedetomidine administered as adjuvants to 0.5% hyperbaric levobupivacaine intrathecally in lower abdominal operations with respect to block characteristics, postoperative analgesia as measured by visual analog scale (VAS) scores, hemodynamic changes, and adverse effects.

Materials and methods

Seventy patients were randomized into two groups of 36 each. Group LF received 15mg of 0.5% hyperbaric levobupivacaine with 25mcg of fentanyl, and Group LD received 15mg of 0.5% hyperbaric levobupivacaine with 10mcg of dexmedetomidine intrathecally.

Results

Patients in Group LD had a significantly longer duration of sensory and motor block than those in Group LF. In Group LF, the onset of sensory and motor blockade was faster (6.25 ± 1.89 minutes and 9.00 ± 3.24 minutes) compared to Group LD (8.70 ± 1.93 minutes and 10.95 ± 4.03 minutes). The length of sensory and motor blockade in Group LD was longer than in Group LF (308.28 ± 6.36 minutes vs. 232.28 ± 7.01 minutes and 198.20 ± 6.52 minutes vs. 157.45 ± 6.30 minutes, respectively). The total postoperative requirement for analgesics in the initial 24 hours, and the mean VAS scores were lower in Group LD.

Conclusion

Patients in Group LD experienced a significantly longer duration of sensory and motor block than those in Group LF. The onset of sensory and motor blockade was significantly faster in Group LF than in Group LD. The total postoperative requirement for analgesics in the initial 24 hours and the mean VAS scores were lower in Group LD. This concludes that the use of dexmedetomidine as an adjuvant to 0.5% hyperbaric levobupivacaine during the subarachnoid block provides prolonged postoperative analgesia, reduces total analgesic requirements, and maintains stable hemodynamics, while the use of fentanyl as an adjuvant to hyperbaric levobupivacaine enhances the onset of both sensory and motor blockade.

## Introduction

Lower abdominal operations are most often conducted under spinal anesthesia since it is economical and fast. The short duration of action of spinal anesthesia using solely local anesthetics makes postoperative pain control difficult; therefore, early analgesic intervention is needed. Various adjuvants, including fentanyl, dexmedetomidine, clonidine, and midazolam, have been investigated to extend the duration of spinal anesthesia. Racemic bupivacaine is one of the most common local anesthetics used for spinal analgesia, and levobupivacaine is its S(-)-enantiomer. Levobupivacaine has been introduced into clinical practice, and its clinical profile has been evaluated in volunteers and clinical studies.

Levobupivacaine blocks sensory and motor function similarly to bupivacaine. The duration of analgesia is prolonged, with rapid recovery from motor block in levobupivacaine. It has a better safety profile, i.e., it has less central nervous system and cardiac toxicity and fewer episodes of hypotension. Opioids exert a synergistic effect with local anesthetics when administered intrathecally. Their use enhances the quality of intraoperative anesthesia, allows for reduced doses of local anesthetics, facilitates a quicker onset of surgical block, and extends the duration of postoperative analgesia [1].

The structures of phenylpiperidine opioids, including fentanyl and meperidine (pethidine), are quite similar to those of local anesthetics. Sensory C primary afferent nerve fibers exhibit a measurable local anesthetic effect from fentanyl, which could contribute to analgesic benefits. The high lipid solubility of these drugs, along with their greater affinity for opioid receptors, results in a more rapid onset of the block, extended postoperative analgesia, and improved quality of intraoperative anesthesia with fewer side effects. Dexmedetomidine, a recent selective α2-agonist, is an effective neuraxial adjuvant for intraoperative and postoperative analgesia with few adverse effects and stable hemodynamics. Previous research suggests that intrathecal 5 μg dexmedetomidine in combination with hyperbaric levobupivacaine in spinal anesthesia may provide sustained postoperative pain relief with minimal side effects [2]. Dexmedetomidine and fentanyl may offer long-lasting postoperative analgesia as adjuvants. Short-acting, lipophilic fentanyl is a potent synthetic opioid analgesic. It has been commonly used as an adjuvant to bupivacaine for postoperative analgesia. In this study, we aim to compare the effects of a combination of levobupivacaine with fentanyl against dexmedetomidine at low doses, focusing on the characteristics of spinal blockade, including onset, duration, hemodynamic parameters, and side effects.

## Materials and methods

"This prospective, randomized trial took place at Pacific Medical College and Hospital, Udaipur, Rajasthan, India, with approval from the institutional ethical committee (ethics clearance number: PMU/PMCH/IEC/PG/2022/156 dated 08/12/2022). It was registered with the Clinical Trials Registry India (CTRI) under registration number CTRI/2024/08/071981. From August to October 2024, this study included 72 individuals aged 18 to 65 years with American Society of Anesthesiologists Physical status (ASA PS) I and II, who were scheduled for elective lower abdominal surgery, including inguinal hernioplasty, appendectomy, and abdominal hysterectomy. Patients were excluded from the study if they had contraindications to spinal anesthesia, obesity (BMI > 30 kg/m²), neuropathy, chronic opioid use, allergy to local anesthetics, or were on beta blockers or had cardiac dysrhythmia. The patients were divided into two equal groups, Group LD and Group LF, through random allocation using sealed opaque envelopes to conceal the allocation sequence. The sealed envelopes were opened by an anesthesiologist responsible for preparing the drug solution as per the randomization but not directly involved in the study process. The anesthesiologist performing the block procedure and observing the study outcomes was unaware of the group treatment, as was the anesthesiologist responsible for data collection. Patients in Group LD were given 15 mg of 0.5% hyperbaric Levobupivacaine combined with 10 mcg of dexmedetomidine in 0.5 ml NS, whereas patients in Group LF received 15 mg of 0.5% hyperbaric Levobupivacaine with 25 mcg of fentanyl in 0.5 ml. The total volume of 3.5 ml was administered intrathecally to each group within 10 seconds. Patients underwent preoperative assessment and fasted for 8 hours, taking Tablet Ranitidine 150 mg and Tablet Alprazolam 0.25 mg the night before surgery. Standard monitoring equipment, such as non-invasive blood pressure, electrocardiography, and pulse oximetry, was set up as soon as the patient arrived in the operating room. A 20-gauge peripheral intravenous cannula was inserted, and the patient received a preload of Ringer's Lactate solution at a rate of 15 ml/kg. Antiemetic prophylaxis was administered with an injection of Ondansetron at a dose of 0.08 mg/kg and an injection of Ranitidine at 1 mg/kg. The patient was positioned sitting upright while maintaining strict aseptic precautions. After disinfecting the skin, 2% lignocaine was infiltrated to provide local anesthesia. A lumbar puncture was then performed at the L3-L4 interspace using a 26-gauge Quincke needle. Once the spinal injection was completed, the patient was immediately assisted into a supine position. The patient was evaluated for sensory and motor block at the following intervals: every 3 minutes for the first 10 minutes, then every 5 minutes for the next 20 minutes, every 10 minutes for 40 minutes, every 20 minutes for 60 minutes, and finally every 30 minutes until the sensory block regressed to the S1 dermatome. The patient was given an intravenous injection of 1 mg midazolam after receiving spinal anesthesia. The patient's vital signs, pulse rate, systolic blood pressure, diastolic blood pressure, mean arterial pressure, and arterial oxygen saturation, were recorded every 5 minutes for the first 15 minutes, every 15 minutes for the next 30 minutes, and every 30 minutes until the surgery was completed. A 3-point scale assessed the sensory block as normal sensation-0, loss of sensation of pinprick (analgesia)-1, and loss of sensation to touch (anesthesia)-2. The time taken for the maximum sensory blockade is the time taken from the injection of the study drug to the maximum sensory blockade attained. The duration of the sensory blockade is the time taken from the injection of the study drug until the patient feels the sensation at the S1 dermatome.

Every minute following the administration of the drug, motor blockage was evaluated using the Modified Bromage Scale (MBS). No power impairment and able to raise the leg-0, Unable to move the hip, able to move the knee, and ankle-1, Unable to move the hip, knee, and able to move the ankle-2, and Unable to move the hip, knee, and ankle-3. The onset of motor blockade is the time taken from the injection of the study drug until the patient is unable to move the hip but able to move the knee and ankle. Patient pain was evaluated by the Visual Analogue Scale (VAS)[3], a scale of zero to ten, where 0 is no pain and 10 is very severe pain. In both groups, the time after administration of the block was considered as time zero. After the block, VAS at time zero served as the baseline score for every patient. If the patient experienced the intensity of pain VAS >4, then an intravenous injection of paracetamol 1 gram 8 hourly was administered as rescue analgesia. Postoperative nausea and vomiting were assessed using the Post-Operative Nausea Vomiting Impact Scale [4]. Scores were assigned based on the episodes of dry retching and nausea as follows: No dry retching and no nausea- scale 0, One episode of dry retching and some nausea-scale 1, Two episodes of dry retching and nausea most of the time- scale 2, and Three or more episodes of dry retching and nausea at all times- scale 3. Utilizing the A.W.A. Crossley and R.P. Mahajan Shivering Score [5], shivering was evaluated, and grades were assigned as follows: no shivering -Grade 0, no visible muscle activity, but one or more piloerections, peripheral vasoconstriction, or peripheral cyanosis (other causes excluded)-Grade 1, muscular activity in a single muscle group -Grade 2, moderate muscular activity in multiple muscle groups without widespread shaking-Grade 3, and violent muscular activity involving the entire body-Grade 4.

Statistical analysis

The sample size for the study was calculated using Kraemer and Thiemann's formula [6], which determined a sample of 36 patients per group. Kraemer and Thiemann formula N=Population Size Z=Critical Value of Normal distribution at the required confidence level P=Sample Proportion E=Margin of Error (Desired Precision) 1-beta=Note that B and power are related to a, the variability of the outcome and the effect size Zn=1.96 for 95% CI, N=(Za+Z1-b)2 P (1-P) / E2, Z1-b=80 for 80% power of study and P=0.5 margin of error (between 5%-95%) E=4.8, Sample Size=72.

Categorical variables were presented in number and percentage (%) and continuous variables were presented as mean and SD. Quantitative variables were compared using the Unpaired t-test as appropriate between the two groups. Qualitative variables were compared using the appropriate Chi-Square test. A p-value of <0.05 was considered statistically significant. The data was entered in MS Ecxel spreadsheet and analyzed using the Statistical Package for Social Sciences (SPSS) version 23.0.

## Results

**Figure 1:**
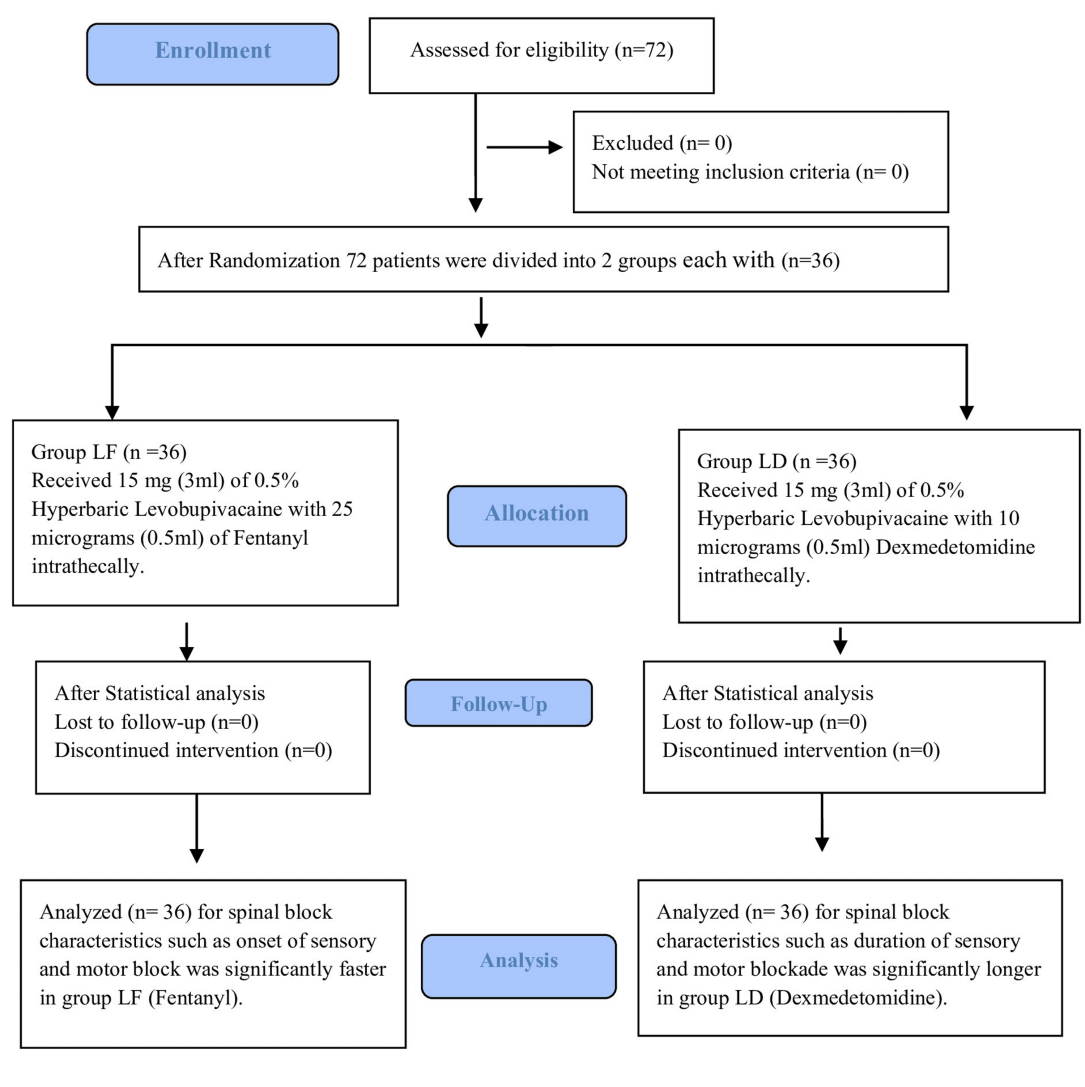
Consolidated standard of reporting trials (CONSORT) flow diagram of study.

This is a prospective, randomized, double-blinded study in which 72 patients were recruited. Patients in Group LD received levobupivacaine with dexmedetomidine, and those in Group LF received fentanyl (Figure 1).

Demographic data are shown in Table 1. There is no statistically significant difference between the groups.

**Table 1:** Demographic characteristics among study groups. Data are presented as mean ± SD, n(%). ASA PS: American Society of Anaesthesiologists Physical status. p<0.05 = significant.

Parameters	Group LF (n=36)	Group LD (n=36)	P-value
Age (years)	44 ± 3	43±4	0.40
BMI (kg/m^2^)	21.81 ± 1.72	21.31 ± 3.05	0.22
Gender (Male/Female)	28/8	21/15	0.45
ASA PS Class I/II	16/20	17/21	0.62
Duration of surgery (Minutes)	90.31 ± 9.05	91.41 ± 11.05	0.32

Block characteristics are shown in Table 2. In Group LD, there was a significantly longer duration of both sensory and motor blockade, but a faster onset of both, with less time required to achieve maximum sensory block compared to Group LF (p<0.01).

**Table 2:** Comparison of sensory and motor block parameters across two groups. Data is expressed in mean±SD. p<0.01 =significant.

Block characteristics	Group LD (n=36) Mean ± SD	Group LF (n=36) Mean ± SD	P-value
Onset of sensory block (in minutes)	8.70 ± 1.93	6.25 ±1.89	< 0.01
Duration of sensory block (in minutes)	308.28 ± 6.36	232.28 ±7.01	< 0.01
Onset of motor block (in minutes)	10.75 ± 4.13	9.02 ± 3.14	< 0.01
Duration of motor block (in minutes	198.20 ± 6.52	157.45 ± 6.30	< 0.01
Time is taken to achieve maximum sensory block (in minutes)	12.25 ± 3.49	14.55 ± 4.86	< 0.01

Hemodynamic parameters were comparable between Group LF and LD, as featured in Table 3 (p>0.05).

**Table 3:** Comparison of hemodynamic parameters among both the groups. Data is expressed in Mean ± SD, p<0.05 =significant.

Time Interval	Hemodynamic Parameters	Group LF (Mean ± SD)	Group LD (Mean ± SD)	t-value	P Value
0 Min	Heart rate	100.53±2.91	99.43±2.49	1.57	0.12
	Systolic Blood Pressure (mm Hg)	133.13±4.96	133.83±5.13	-0.53	0.60
	Diastolic Blood Pressure (mm Hg)	83.53±3.91	85.20±5.66	-1.32	0.20
	Mean Arterial Pressure (mm Hg)	100.00±3.24	100.70±5.81	-0.57	0.56
5 Min	Heart rate	97.30±4.24	97.93±3.65	-0.62	0.53
	Systolic Blood Pressure (mm Hg)	129.10±6.09	132.20±7.70	-1.72	0.09
	Diastolic Blood Pressure (mm Hg)	81.50±6.16	84.27±6.46	-1.69	0.09
	Mean Arterial Pressure (mm Hg)	97.17±5.05	99.70±7.41	-1.54	0.12
10 Min	Heart rate	85.70±4.53	84.63±4.24	0.94	0.35
	Systolic Blood Pressure (mm Hg)	116.47±6.15	118.77±6.90	-1.36	0.17
	Diastolic Blood Pressure (mm Hg)	72.23±5.73	72.87±6.49	-0.401	0.69
	Mean Arterial Pressure (mm Hg)	86.53±4.80	87.73±5.78	-0.87	0.38
15 Min	Heart rate	80.50±5.96	77.93±5.13	3.18	0.07
	Systolic Blood Pressure (mm Hg)	110.60±7.08	112.10±7.27	-0.80	0.42
	Diastolic Blood Pressure (mm Hg)	68.70±5.29	67.60±5.88	0.761	0.44
	Mean Arterial Pressure (mm Hg)	82.30±5.55	81.67±6.47	0.40	0.68
30 Min	Heart rate	73.27±6.56	70.57±5.30	4.35	0.08
	Systolic Blood Pressure (mm Hg)	103.03±3.15	102.17±5.89	0.71	0.48
	Diastolic Blood Pressure (mm Hg)	62.27±6.52	60.87±5.29	0.913	0.36
	Mean Arterial Pressure (mm Hg)	75.87±4.78	74.80±4.45	0.894	0.37
45 Mins	Heart rate	71.50±6.15	70.20±4.13	5.39	0.34
	Systolic Blood Pressure (mm Hg)	103.20±3.29	102.67±5.54	0.453	0.652
	Diastolic Blood Pressure (mm Hg)	62.13±4.91	60.17±4.85	1.561	0.12
	Mean Arterial Pressure (mm Hg)	75.73±4.08	74.00±4.04	1.654	0.10
60 minutes	Heart rate	69.13±6.25	67.03±3.98	5.24	0.12
	Systolic Blood Pressure (mm Hg)	103.75±2.19	104.88±7.06	-0.43	0.67
	Diastolic Blood Pressure (mm Hg)	62.00±5.07	62.38±6.12	-0.128	0.90
	Mean Arterial Pressure (mm Hg)	75.71±3.90	75.25±5.18	0.194	0.84
90 Minutes	Heart rate	64.00±2.31	64.12±1.46	-0.12	0.90
	Systolic Blood Pressure (mm Hg)	113.90±4.82	111.77±3.66	2.08	0.06
	Diastolic Blood Pressure (mm Hg)	72.37±4.04	71.63±3.06	0.79	0.43
	Mean Arterial Pressure (mm Hg)	85.93±2.16	84.67±3.48	1.69	0.09
120 Minutes	Heart rate	73.00±6.24	72.13±5.99	0.54	0.58
	Systolic Blood Pressure (mm Hg)	127.77±2.98	126.67±3.59	1.29	0.20
	Diastolic Blood Pressure (mm Hg)	75.27±5.11	77.27±5.25	-1.49	0.14
	Mean Arterial Pressure (mm Hg)	91.83±2.18	92.60±2.67	-1.22	0.30

**Figure 2:**
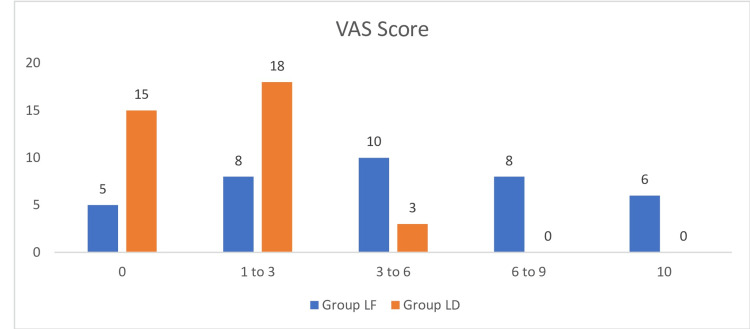
Comparison of VAS scores among study groups. VAS: Visual Analogue Scale.

The VAS [3] was used to assess analgesia in the two study groups. A significantly lower VAS score was noted in Group LD compared to Group LF (p<0.01, Figure 2).

**Figure 3:**
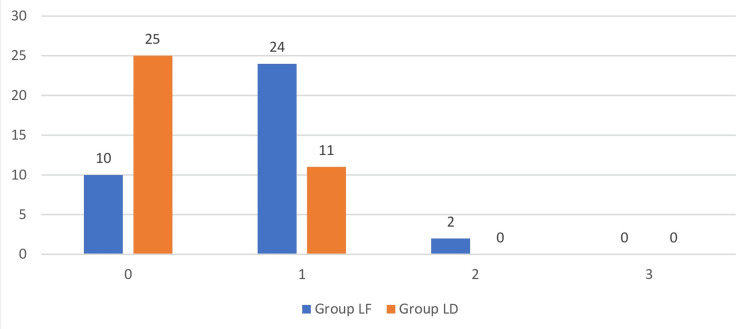
Comparison of post operative nausea and vomiting among groups. PONV: Post Operative Nausea and Vomiting.

There was a significantly lower incidence of Post-Operative Nausea Vomiting Impact Scale Score in Group LD compared to Group LF (p<0.05) (Figure 3).

**Figure 4:**
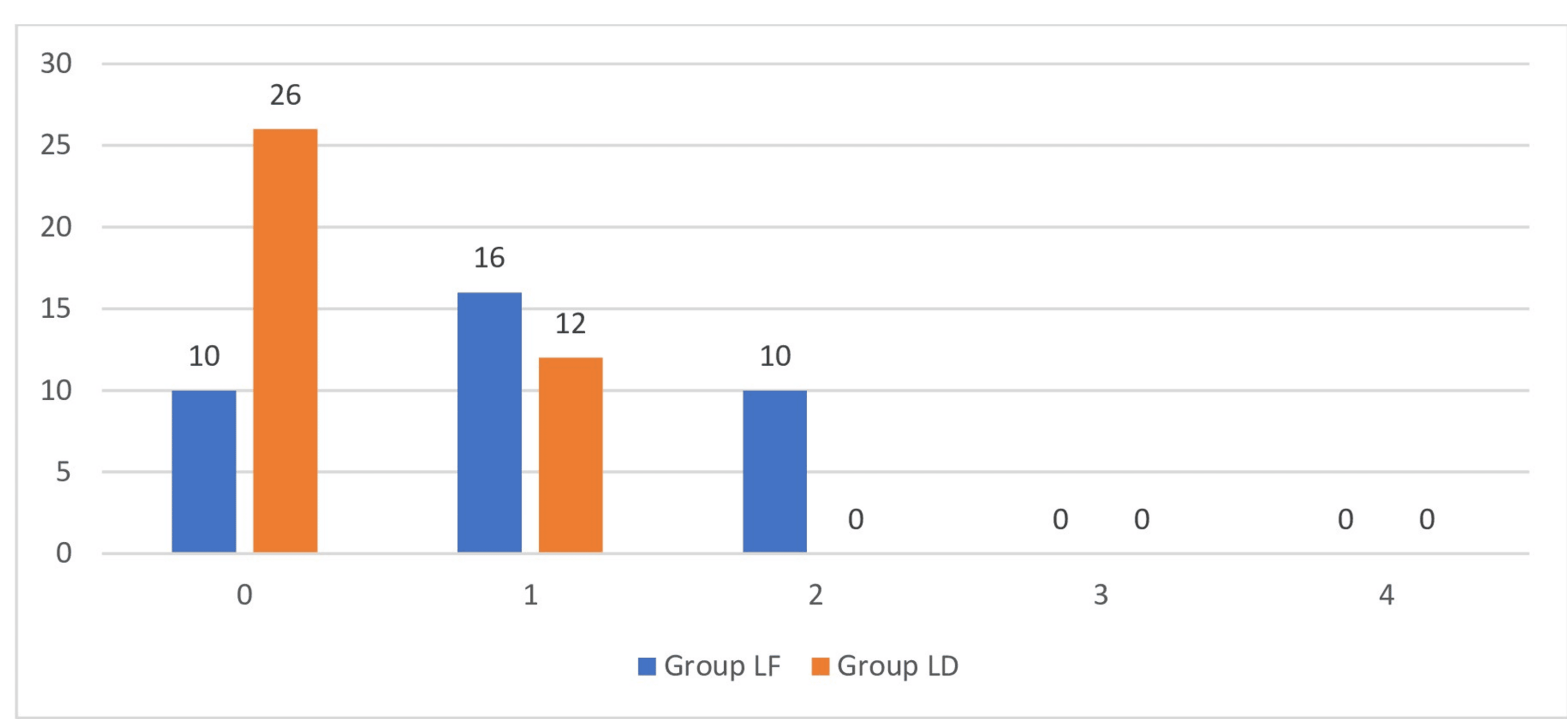
Comparison of post operative shivering among groups.

The A.W.A. Crossley and R.P. Mahajan Shivering Score was used to assess shivering among the study groups. There was a significantly lower incidence of post-operative shivering in Group LD compared to Group LF (p<0.05) (Figure 4).

The requirement for rescue analgesia was significantly lower in Group LD compared to Group LF (p<0.05).

## Discussion

The long-acting, amide-type local anesthetic levobupivacaine, the S(−) 3-isomer of racemic bupivacaine, offers prolonged anesthesia in a dose-dependent manner. It acts within 15 minutes using various anesthetic techniques and administration routes, such as epidural, intrathecal, and peripheral nerve blocks. In our study, we used 15 mg of hyperbaric levobupivacaine with adjuvants such as dexmedetomidine and fentanyl for patients planning lower abdominal surgeries, achieving faster onset and shorter duration with fentanyl, 6.25 ± 1.89 minutes and 232.28 ± 7.01 minutes, respectively. However, there was a longer sensory blockade compared to the shorter motor blockade with dexmedetomidine, 308.28 ± 6.36 minutes and 198.20 ± 6.52 minutes, respectively. Postoperative pain is well managed with intrathecal hyperbaric bupivacaine with dexmedetomidine compared to fentanyl. Research on surgical anesthesia in adults found that levobupivacaine provided sensory block for up to 9 hours following epidural treatment (≤202.5 mg), 6.5 hours after intrathecal 15 mg, and 17 hours after brachial plexus block (2 mg/kg). Levobupivacaine caused sensory block longer than bupivacaine, lasting 23 to 45 minutes with epidural administration and 2 hours with peripheral nerve block. Levobupivacaine epidural motor block was shorter than the sensory block. This differential was not seen with peripheral nerve block. The recommended maximum single dose for intrathecal administration is 15 mg [7].

In our study, the duration of sensory block was longer in Group LD (308.28 ± 6.36 minutes) compared to Group LF (232.28 ± 7.01 minutes), findings concordant with those of Khosravi F et al. [8] and Varghese et al. [9]. Dexmedetomidine produces longer motor and sensory blocks than fentanyl intrathecal to isobaric bupivacaine in vaginal hysterectomy, according to Al-Ghanem et al. [10]. In our investigation, dexmedetomidine caused extended sensory and motor blockage, stable hemodynamics, and patient satisfaction. Al-Mustafa et al. observed that dexmedetomidine 5 and 10 μg with bupivacaine in urological procedures prolongs spinal anesthesia duration in a dose-dependent manner [11].

Lee et al. noted that 0.5% levobupivacaine with fentanyl is as effective as 0.5% alone in urological surgery in terms of clinical efficacy, motor block, and hemodynamic effects. This suggests that adding fentanyl to levobupivacaine reduces motor block duration, 157.45 ± 6.30 minutes, but does not affect hemodynamic stability, as demonstrated in our study [12].

Bidikar et al. conducted a comparison of levobupivacaine 0.5% with a combination of levobupivacaine and fentanyl, revealing that the latter extended the duration of sensory block and delayed the requirement for additional analgesia, while not prolonging motor block. The hemodynamics exhibited similarity across both groups. The clinical significance of a reduced duration of motor block due to lower-dose levobupivacaine combined with fentanyl is early ambulation, consistent with our study findings [13].

Sinchana AS et al. compared Intrathecal Hyperbaric Levobupivacaine with Fentanyl to Hyperbaric Bupivacaine with Fentanyl in elective cesarean procedures. They discovered that the levobupivacaine group had fewer side effects and much shorter periods of sensory and motor block than the bupivacaine group. Levobupivacaine causes analgesia for a substantially shorter time than bupivacaine. In our study, sensory blockage was comparable to the fentanyl group [14].

Xia F et al. demonstrated that the addition of dexmedetomidine to local anesthetic as an adjuvant in a central neuraxial block prolongs the duration of analgesia. They concluded that intrathecal dexmedetomidine enhanced the antinociceptive effects of hyperbaric bupivacaine by 31% in spinal anesthesia for patients undergoing cesarean section. In our study, the incorporation of dexmedetomidine with levobupivacaine also extends the duration of analgesia [15].

In our study, the incidence of perioperative shivering was significantly lower with dexmedetomidine, concordant with findings by Zhang J et al. [16]. ShuJun S et al., compared to fentanyl, found that dexmedetomidine as an adjuvant to local anesthetics in spinal anesthesia prolonged the duration of spinal anesthesia and improved postoperative analgesia, similar to our study [17].

## Conclusions

In this randomized study, we assessed the effectiveness of intrathecal fentanyl (25 mcg) and dexmedetomidine as adjuncts to 0.5% hyperbaric levobupivacaine in patients undergoing lower abdominal surgeries with spinal anesthesia. Our results showed that dexmedetomidine significantly prolonged postoperative analgesia compared to fentanyl, although fentanyl had a quicker onset of the sensory block. Both additives had minimal impact on vital signs, indicating they are safe and effective adjuncts. Notably, dexmedetomidine offered a longer block duration, reduced analgesic needs, and lower rates of postoperative shivering, nausea, and vomiting, along with lower VAS scores than fentanyl.
